# Assessment of Heavy Metal Pollution and Health Risks in the Soil-Plant-Human System in the Yangtze River Delta, China

**DOI:** 10.3390/ijerph14091042

**Published:** 2017-09-10

**Authors:** Bifeng Hu, Xiaolin Jia, Jie Hu, Dongyun Xu, Fang Xia, Yan Li

**Affiliations:** 1 Institute of Agricultural Remote Sensing and Information Technology Application, Zhejiang University, Hangzhou 310029, China; hubifeng@zju.edu.cn (B.H.); 21514120@zju.edu.cn (X.J.); jiehu@zju.edu.cn (J.H.); xdy900815@126.com (D.X.); maggie_xia@263.net (F.X.); 2 Institute of Land Science and Property, School of Public Affairs, Zhejiang University, Hangzhou 310058, China

**Keywords:** heavy metals, soil-plant-human systems, health risk assessment, hazard quotients, carcinogens risk, bioaccumulation factors

## Abstract

Heavy metal (HM) contamination and accumulation is a serious problem around the world due to the toxicity, abundant sources, non-biodegradable properties, and accumulative behaviour of HMs. The degree of soil HM contamination in China, especially in the Yangtze River Delta, is prominent. In this study, 1822 pairs of soil and crop samples at corresponding locations were collected from the southern Yangtze River Delta of China, and the contents of Ni, Cr, Zn, Cd, As, Cu, Hg, and Pb were measured. The single pollution index in soil (SPI) and Nemerow composite pollution index (NCPI) were used to assess the degree of HM pollution in soil, and the crop pollution index (CPI) was used to explore the degree of HM accumulation in crops. The bioaccumulation factor (BAF) was used to investigate the translocation of heavy metals in the soil-crop system. The health risks caused by HMs were calculated based on the model released by the U.S. Environmental Protection Agency. The SPIs of all elements were at the unpolluted level. The mean NCPI was at the alert level. The mean CPIs were in the following decreasing order: Ni (1.007) > Cr (0.483) > Zn (0.335) > Cd (0.314) > As (0.232) > Cu (0.187) > Hg (0.118) > Pb (0.105). Only the mean content of Ni in the crops exceeded the national standard value. The standard exceeding rates were used to represent the percentage of samples whose heavy metal content is higher than the corresponding national standard values. The standard exceeding rates of Cu, Hg, and Cd in soil were significantly higher than corresponding values in crops. Meanwhile, the standard exceeding rates of Ni, As, and Cr in crops were significantly higher than corresponding values in soil. The chronic daily intake (CDI) of children (13.8 × 10^−3^) was the largest among three age groups, followed by adults (6.998 × 10^−4^) and seniors (5.488 × 10^−4^). The bioaccumulation factors (BAFs) of all crops followed the order Cd (0.249) > Zn (0.133) > As (0.076) > Cu (0.064) > Ni (0.018) > Hg (0.011) > Cr (0.010) > Pb (0.001). Therefore, Cd was most easily absorbed by crops, and different crops had different capacities to absorb HMs. The hazard quotient (HQ) represents the potential non-carcinogenic risk for an individual HM and it is an estimation of daily exposure to the human population that is not likely to represent an appreciable risk of deleterious effects during a lifetime. All the HQs of the HMs for the different age groups were significantly less than the alert value of 1.0 and were at a safe level. This indicated that citizens in the study area face low potential non-carcinogenic risk caused by HMs. The total carcinogens risks (TCRs) for children, adults, and seniors were 5.24 × 10^−5^, 2.65 × 10^−5^, and 2.08 × 10^−5^, respectively, all of which were less than the guideline value but at the alert level. Ingestion was the main pathway of carcinogen risk to human health.

## 1. Introduction

**Figure 1:**
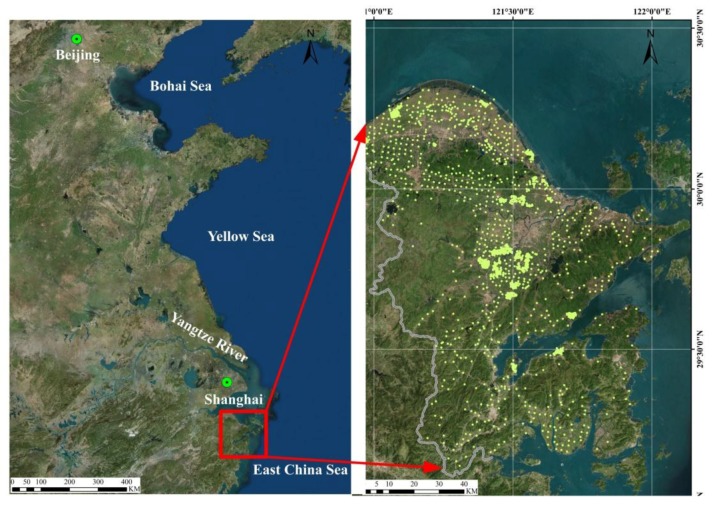
Location of the study area and sampling points.

Heavy metal (HM) contamination and accumulation is a serious problem around the world due to the potential threat to food safety and its detrimental effects on human and animal health [1,2,3,4]. It has also become one of the major environmental problems in China due to continuous industrialization and urbanization [5,6]. According to the National Soil Pollution Condition Investigation Communique released by the Ministry of Land and Resources and the Ministry of Environmental Protection of the People’s Republic of China, the proportion of HM contaminated samples in China is 16.1% [7]). The pollution degree of soil HM contamination in the Yangtze River Delta is remarkably high [8]. The selected study area is an important coastal industrial city located on a typical flat alluvial plain in the Yangtze River Delta (YRD) region, the most developed economic district in eastern China (see Figure 1). There are more than 100 million people living in the Yangtze River Delta [9]. Therefore, it is very necessary to explore the state of HM pollution in soil and agricultural food and to assess the potential health risk caused by HM pollution.

Many researchers have found that HMs are easily accumulated in various edible vegetables and fruits through contaminated soil [10,11,12,13]. Satsananan (2012) reported that lead (Pb) and cadmium (Cd) accumulated in basil, ginger, turmeric, lemon grass, parsley, onion, and coriander glory [14]. HMs in soil can threaten human health through vegetable consumption, and the chronic low-level intake of soil metals through ingestion or inhalation has a seriously negative effect on human health [15,16,17]. For example, chronic exposure to Cd can have harmful effects, such as lung cancer, prostatic proliferative lesions, bone fractures, kidney dysfunction, and hypertension [18]. The chronic effects of As include bladder cancer, kidney cancer, skin cancer, lung cancer, and liver cancer [19,11]. Exposure to lead (Pb) may cause plumbism, anaemia, nephropathy, gastrointestinal colic, and central nervous system symptoms [20]. When HMs are transferred into food chains and accumulate in vital organs, such as the liver, kidneys, and bones, there is a direct threat to human health [21] that can result in numerous serious health disorders [22]. Vegetables which are heavily contaminated by HMs may cause gastrointestinal cancer and heart disease, damage the memory and intellectual abilities of human beings, disrupt numerous biochemical processes, and lead to cardiovascular, nervous, kidney, and bone diseases [23]. The gross domestic product (GDP) of the Yangtze River Delta (YRD) region accounts for 16.7% of China’s GDP [24]. Due to the dramatically increasing industry operations and rapid urban expansion in the past three decades, the soil has been subjected to HM contamination due to increasing pollutant inputs from anthropogenic sources [25]. This is the first time that the HM pollution condition in the soil-plant system has been assessed and the potential health risks of exposure identified in such a critical region of China. The results of this study can provide a reference for some other countries (such as India, Brazil, and Iran) which are accelerating industrialization. Furthermore, since grains, tubes, vegetables, beans, fruit, and teas are widely consumed around the world, research results about bioaccumulation of HMs in crops can create benefits for many related research studies in other areas. Therefore, it has great significance to investigate the contamination of HMs in the soil-plant-human system and to identify the health threat of HMs to citizens in the YRD.

Traditional laboratory analyses of heavy metals in soils, such as Atomic Fluorescence Spectrometry (AFS), Atomic Absorption Spectrometry (AAS), and Inductively Coupled Plasma Optical Emission Spectroscopy (ICP-OES), are time consuming, laborious, and expensive. Some other methods such as portable X-ray fluorescence (PXRF) [4], Laser-induced Breakdown Spectroscopy (LIBS) [26], hyperspectral [27], visible-near infrared spectroscopy (Vis-NIR), and mid-infrared spectroscopy (MIR) [28] have been considered as rapid, effective techniques to measure total concentrations of heavy metals in soil. Recently, the combined use of Vis-NIR, MIR, and PXRF technology has also shown bright promise for predicting heavy metal content in soil [28]. However, traditional laboratory analysis methods have higher accuracy compared with PXRF and LIBS. Therefore, in this paper we used traditional laboratory analysis methods according to national standard [29]. A comprehensive understanding of the HM pollution in soil-plant systems and potential health risk of HMs in soil-plant systems is essential in order to make informed decisions on the approaches to reduce contamination, minimize human exposure, and protect populations from the risk. Assessments of HM contamination and health risks in contaminated soil and vegetables have been conducted in several countries [17,4], but few studies have been performed to assess the pollution grade of HMs in a soil-food-human chain and the health risks caused by HM in soil. Therefore, the main objectives of this study are as follows: (1) determine the contamination levels of eight HMs, including chromium (Cr), lead (Pb), cadmium (Cd), mercury (Hg), arsenic (As), copper (Cu), zinc (Zn), and nickel (Ni), in soil and plants; (2) evaluate the potential health risks caused by HMs in different age groups via different pathways in the Yangtze River Delta, China; (3) analyse the bioaccumulation factor of heavy metals in soil-plant systems.

## 2. Materials and Methods

### 2.1. Study Area

The selected study area is an important coastal industrial city which is located on a typical flat alluvial plain in the Yangtze River Delta (YRD) region (28°51′–30°33′ N, 120°55′–122°16′ E) [30], the most developed economic district in eastern China (see Figure 1). The study area is located in a subtropical region; the climate is mild and humid, with an annual average temperature of 16.4 °C and an annual precipitation of 1480 mm. The study area covers 9816 km^2^ and has a population of 7.81 million. Due to rapid industrialization, HM pollution in paddy fields in the study area is of increasing concern. Moreover, this city is an important chemical industrial base in China. The chemical, textile and garment, and machinery industries are three industrial pillars. Petrochemical, electronic, metallurgy, engineering, building materials, and textile industries have also been developed. However, due to the dramatically increased industrial operations and rapid urban expansion in the past three decades, the soil environment is faced with HM contamination due to increasing pollutant inputs from anthropogenic sources [25].

### 2.2. Sampling and Chemical Analysis

A total of 1822 pairs of soil and crop samples were collected from the study area. Systematic grid sampling was applied. At some of the grid nodes, grid sampling was augmented by sampling nearby areas. Each soil sample was combined with five subsamples collected from five locations within five meters. All soil subsamples were collected at a depth of 0–20 cm using a stainless steel shovel. During the harvest season, 1822 corresponding crop samples were collected at the same locations as soil samples at different maturation times. The crop samples included 933 grain samples, 650 vegetable samples, 117 fruit samples, 92 bean samples, 24 tuber samples, and seven tea samples. The coordinates of the sampling locations were recorded using a differential global positioning system (GPS) (see Figure 1).

Soil samples were air-dried in the laboratory for several days at ambient temperature and were passed through a 2-mm nylon sieve for a general analysis of the soil properties. Then, some of the soil samples were ground to pass through 100 meshes and stored in closed polyethylene bags for HM content analysis. Crop samples were oven-dried at 105 ℃ for 1 h and then at 70 ℃ to constant weight. Then, crop samples were comminuted using a pulveriser, ground to pass through 100 meshes using a nylon sieve, and stored in closed polyethylene bags for further analysis.

Soil pH was measured in H_2_O with a soil/solution ratio of 1:2.5 (*m*/*v*), using the Glass Electrode method (GL, pHS-3C, REX, Shanghai, China) according to the agricultural sector standard (NY/T 1377–2007) of the People’s Republic of China. The total concentrations of Cr, Pb, As, Cu, Zn, and Ni in soil samples were acid-digested with HCl–HNO_3_–HClO_4_, and the crops samples were digested by means of the dry ashing method [31], and determined using inductively coupled plasma optical emission spectrometry (ICP-OES 6300, Thermo Fisher Scientific, Waltham, MA, USA). Total Cd in the soils and vegetables were digested by HF–HNO_3_–HClO_4_ and analysed by an inductively coupled plasma-mass spectrometer (ICP-MS, Agilent 7500a, Palo Alto, CA, USA). Total Hg in soil and plant were digested by HNO_3_–HCl in a water bath and determined by a double channel Atomic Fluorescence Spectrometer. Reagent blanks and standard reference materials were used in the analysis for quality assurance and quality control. The recoveries of the elements ranged from 90 to 110%.

### 2.3. Method of Soil Heavy Metal Pollution Assessment

The degree of soil HM pollution was assessed as follows: first soil pH values were categorized into three classes: <6.5, 6.5 ≤ pH ≤ 7.5, and >7.5; second, the pollution threshold for each soil HM was determined by land use (e.g., paddy fields) and pH class; third, the single pollution index (SPI) for each HM was determined (Equation (1)); finally, the Nemerow composite pollution index (NCPI) was calculated (Equation (2)).

(1) $$P_{i}=\frac{C_{i}}{S_{i}}$$

where $C_{i}$ is the concentration of soil HM *i*, and *S_i_* is the pollution threshold of i [32].

(2) $$NCPI=\sqrt{\frac{(P_{imax}{)}^{2}+({\bar{P}}_{i}{)}^{2}}{2}}$$

where $P_{\mathrm{imax}}$ is the maximum SPI value of each HM and $\bar{P}$ is the mean SPI of each HM [32].

As NCPI is a comprehensive index, it was used to classify the soils in terms of HM pollution. Table 1 shows the classifications of the single pollution index (SPI) and Table 2 shows the classifications of Nemerow composite pollution index (NCPI).

### 2.4. Method of Crop Heavy Metal Pollution Assessment

The pollution levels of HMs in crops were evaluated using the crop pollution index (CPI). The CPI was defined as the ratio of an element concentration in the crop samples to the national standard value of the corresponding element of the national hygienic standards for food in China. The CPI was calculated using Equation (3):

(3) $$CPI_{i}=\frac{CC_{i}}{CS_{i}}$$

where ${\mathrm{CC}}_{i}$ represents the measured concentration of element *i*, and ${\mathrm{CS}}_{i}$ is the national standard value of element *i* according to the national hygienic standard for food in China (Table 3).

The bioaccumulation factor (BAF) of each crop was used to assess the transfer of HMs from soil to plant. It can be calculated as:

(4) $$BAF=\frac{C_{C}}{C_{S}}$$

where $C_{C}$ and $C_{S}$ are the total HM concentrations in some kind of crops and corresponding soils samples, respectively [34], when calculating the BAF of some kind of crops (food, vegetables, fruits, beans, and tubers). When calculating the overall BAF, $C_{C}$ indicates the mean content of heavy metals in all crops samples while $C_{S}$ represents the mean content of corresponding heavy metals in all soil samples.

### 2.5. Health Risk Assessment of Heavy Metals in Soils

Health risk assessment, including non-carcinogenic and carcinogenic risk assessment via three exposure pathways: ingestion, dermal contact, and inhalation, has been recognized as an important tool for identifying health risk in human activities and providing risk evidence for decision-makers [35]. The methodology used for the health risk assessment was based on the guidelines and Exposure Factors Handbook released by the U.S. Environmental Protection Agency [36,37,38]. Due to their behavioural and physiological differences, in this study, the population was divided into three groups—children, adults, and seniors—and the exposure paths were divided into three paths: inhalation, dermal contact, and ingestion.

Chronic daily intake (CDI, mg/kg/day) was used to evaluate exposure to HMs in the soil. Direct exposure to soil was estimated through three pathways: (1) inhalation of particulates emitted from the soil, (2) dermal contact with the soil, and (3) incidental ingestion of the soil. The CDI of the three exposure pathways was defined using U.S. Environment Protection Agency (USEPA) methodology [39,40]. The three equations are as follows [6]:

(5) $${\mathrm{CDI}}_{Inhalation}=\frac{PM_{10}\times M_{PM}\times ET\times IR_{air}\times EF\times ED}{BW\times AT\times PEF}$$

(6) $${\mathrm{CDI}}_{Dermal}=\frac{C_{soil}\times SA\times PE\times AF\times ABS\times ED}{BW\times AT\times 10^{6}}$$

(7) $${\mathrm{CDI}}_{Ingestion}=\frac{C_{soil}\times IR_{soil}\times EF\times ED}{BW\times AT\times 10^{6}}$$

where PM_10_ is the ambient particulate matter in a similar area in the YRD region (0.146 mg/m^3^) [41]; M_PM_ is the HM concentration of airborne particulate matter, assumed to be equal to C_soil_, where dust is derived from the soils [42]; *ET* is the exposure time (hours/day); IR_air_ is the inhalation rate of air (m^3^/day); *EF* is the exposure frequency (days/year); *ED* is the exposure duration (year); C_soil_ is the concentration of HMs in the soil (mg/kg); *SA* is the skin surface area for soil contact (cm^2^/day); *FE* is the fraction of dermal exposure ratio to the soil; AF is the soil adherence factor (mg/cm); ABS is the fraction of applied dose absorbed across the skin; and 10^6^ is the conversion factor from kg to mg. Body-function parameters, such as body weight (*BW*), were taken from the National Physique Monitoring Bulletin 2014 [7]. Other exposure variables were obtained from the USEPA Integrated Risk Information System. The CDI of HMs for children (3–12 years old), adults (18–45 years old), and seniors (>45 years old) were calculated separately. The parameters were provided by the USEPA [43,40].

The hazard quotient (HQ) represents the potential non-carcinogenic risk for an individual HM. The HQ is defined as the ratio of CDI (mg/kg/day) to the reference dose (RfD, mg/kg/day) and is an estimation of daily exposure to the human population that is not likely to represent an appreciable risk of deleterious effects during a lifetime [40]:

(8) $$HQ=\frac{CDI}{RfD}$$

(9) $$HI=\sum_{i=1}^{n}HQ_{i}=HQ_{Inhalation}+HQ_{Dermal}+HQ_{Ingestion}$$

The values of RfD for the selected heavy metals in the different exposure pathways are provided by the USEPA [43,40]. With respect to the assessment of the overall potential risk posed by more than one HM, HQs can be added to generate a hazard index (HI) to estimate the combined risk (Equation (9)) [37]. If HI exceeds 1.0, there is a chance that non-carcinogenic effects will occur, and the probability tends to increase with the value. Otherwise, there are likely to be no non-carcinogenic effects.

For carcinogens, risk is estimated as the incremental probability of an individual developing cancer over a lifetime as a result of exposure to the potential carcinogenic risk [44]. Potential carcinogenic risk can be evaluated using the following equations [6]:

(10) CR=CDI×CSF

(11) $$TCR=\sum_{i=1}^{n}CDI_{i}\times CSF_{i}$$

where CR is the probability of carcinogenic risk (dimensionless), TCR is the total probability of carcinogenic risk, and CSF is the carcinogenic slope factor of each metal (1/mg/kg/day). Total carcinogenic risk is equal to the sum of the risk from all exposure pathways from all individual metals. The values of CSF for the selected heavy metals in different exposure pathways are provided by the USEPA [40]. The range of acceptable total risk for regulatory purposes is 10^–6^ to 10^–4^ [40,45]. In regulatory terms, a TCR less than or equal to 10^–6^ represents virtual safety, and a TCR equal to or greater than 10^–4^ indicates a potentially great risk [43].

### 2.6. Data Analysis

Statistical analysis of the data was performed using Origin 8 (Origin 8 SR4, Northampton, MA, USA) and Microsoft Excel 2010 (Office 2010, Redmond, WA, USA). ArcGIS10.3 software (ESRI, ArcGIS 10.3, Redlands, CA, USA) was used to map the sampling sites.

## 3. Results and Discussion

### 3.1. Descriptive Statistics of Heavy Metals in Soil and Plants

The descriptive statistics (such as mean value, median value, coefficients of variation (CV) and standard deviation (Std)) of the HM contents in the soil are presented in Table 4. The CV of Hg—is greatest. This showed that there was great heterogeneity in the content of Hg in the soil across the study area, indicating that anthropogenic inputs may be the main sources of Hg in this area [42,35]. The maximum content of Cr, Pb, Cd, Hg, Cu, Zn, and Ni were significantly higher than the critical level of Environmental Quality Standards for the soils in China. Thus, these elements require intensive monitoring to prevent further accumulation.

The descriptive statistics of the HM contents in crops are summarized in Table 5. The concentrations of Cr, Pb, Cd, Hg, As, Cu, Zn, and Ni ranged from 0.01 to 13.00, 0.01 to 1.50, 0.01 to 1.10, 0.01 to 0.024, 0.10 to 21.00, 0.39 to 56.00, and 0.01 to 7.80 mg/kg, respectively, with mean contents of 0.44, 0.05, 0.05, 0.02, 0.16, 1.97, 14.22, and 0.39 mg/kg, respectively (Table 5). The main HMs in crops were not consistent with the HMs in the soils (Table 4), indicating accumulation differences between HMs in different crops and suggesting that the absorption may be affected by other factors such as HM concentration in soils [47,48,49]. The content of Cr, Pb, Cd, Hg, As, and Ni in crops had extensive variability, with coefficients of variation of 170.45%, 180.00%, 120.00%, 120.80%, 256.25%, and 125.64%, respectively, whereas Cu and Zn showed moderate variability, with coefficients of variation of 79.70% and 76.86%, respectively. According to the guidelines in China (Table 3), the mean concentrations of Cr, Pb, Cd, and Hg were higher than the maximum allowable levels in food. The mean concentrations of As and Cu were lower than the maximum allowable levels in food. The mean concentration of Ni was lower than the maximum allowable level in grain but higher than that in other foods. This indicated that measures need be taken to prevent further accumulation of Cr, Pb, Cd, and Hg in crops.

### 3.2. Assessment of Heavy Metals in Soils and Plants

According to the soil quality standards of China [29], Class II can be used as the threshold value for human health protection. To substantiate the soil pollution caused by heavy metals, the percentages of soil samples in comparison with the environmental quality standard for soils in China (GB15618-1995) [29] were calculated.

The descriptive summary of the SPI and NCPI of the HM contents in the soils is presented in Table 6. The mean SPIs of different elements were in the following decreasing order: Hg (0.948) > Ni (0.670) > Cd (0.585) > Zn (0.508) > Cu (0.438) > Cr (0.321) > As (0.229) > Pb (0.162). The SPIs of all elements were at the safety level. The mean NCPI was 0.846 and at the alert level'.

The SPIs of each of the HMs was calculated to assess the pollution degree of different HMs in soils and it was classified according to Table 1. The pollution grade classification of the SPIs in soil samples is shown in Table 7. As shown in Table 7, 0.60% of the Cr samples, 0.05% of the Pb samples, 7.14% of the Cd samples, 33.32% of the Hg samples, 4.12% of the Cu samples, 1.37% of the Zn samples, and 7.96% of the Ni samples in the soil exceeded their Grade II values (Table 7). According to the above analysis, Hg, Ni, and Cd soil contamination levels were relatively highly, among which, Hg contamination was the most serious.

The crop CPIs were calculated according to Equation (3) and then their descriptive statistics (which include mean, median, standard deviation (Std), Min, Max, and CV, all of which are summarized in Table 8), the number of polluted samples, and the corresponding percent were also calculated. The mean CPIs were in the following decreasing order: Ni (1.007) > Cr (0.483) > Zn (0.335) > Cd (0.314) > As (0.232) > Cu (0.187) > Hg (0.118) > Pb (0.105). This order was different from that of the SPIs in soil, indicating different accumulation capacities of the crops for these heavy metals. As shown in Table 8, the percent of polluted samples (that is, the percent of plant samples in which the HM content exceeded the national standard) for Cr, Pb, Cd, Hg, As, Cu, Zn, and Ni were 11.30%, 0.44%, 3.24%, 0.11%, 5.70%, 0.05%, 0.66%, and 37.96%, respectively. These numbers were significantly different from those in soil, indicating that many other factors, such as soil pH [50], organic matter [51], and phosphorous content [52], might influence metal uptake by crops.

### 3.3. Human Health Risk Assessment of Heavy Metals in Soils

#### 3.3.1. Exposure Analysis

**Figure 2:**
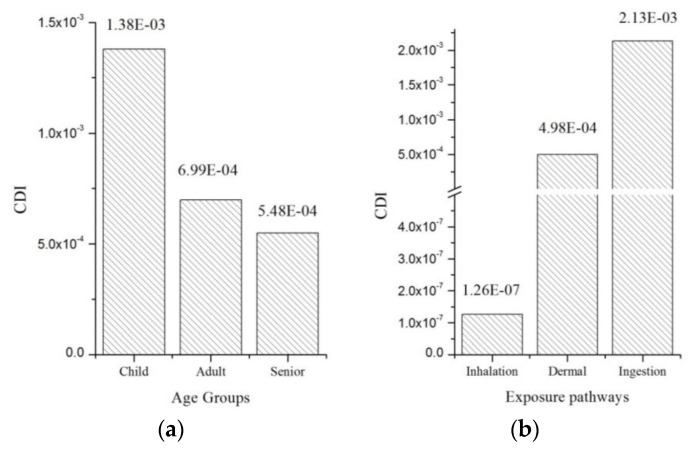
Total CDIs (**a**) and (**b**) of different exposure pathways in different age groups.

As shown in Figure 2, the CDIs due to eight heavy metals in the study area for different age groups (seniors, adults, and children) were evaluated under different exposure pathways (ingestion, inhalation, and dermal contact). The CDI for children was the largest among the three age groups (13.8 × 10^–3^), followed by adults (6.99 × 10^–4^) and seniors (5.48 × 10^–4^) (Figure 2a). The CDIs of the exposure pathways were in the order of ingestion (2.13 × 10^–3^) > dermal contact (4.98 × 10^–4^) > inhalation (1.26 × 10^–7^) (Figure 2b). This revealed that ingestion was the main pathway through which citizens in the study area are exposed to HMs.

#### 3.3.2. Non-Carcinogenic Risk Assessment

**Figure 3:**
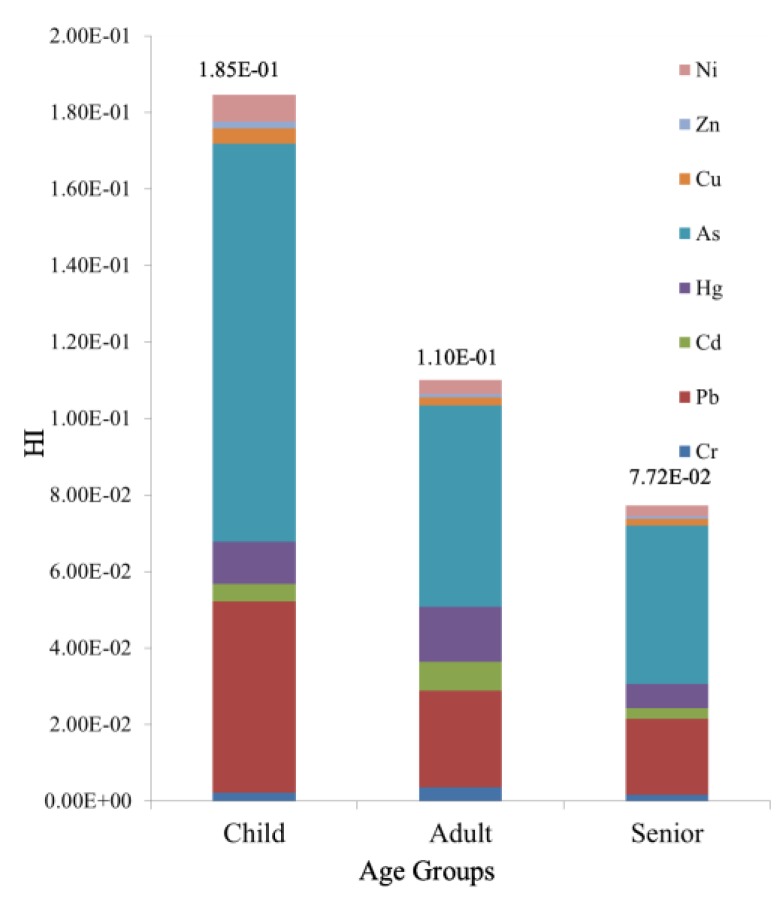
Hazard indices (His) of different age groups.

The HIs for eight HMs (Cr, Pb, Cd, Hg, As, Cu, Zn, and Ni) in the study area for different age groups (adults and children) were evaluated under different exposure pathways (ingestion, inhalation, and dermal contact). As shown in Figure 3, the HIs were in the following decreasing order: children (1.85 × 10^–1^) > adults (1.10 × 10^–1^) > seniors (7.72 × 10^–2^). The HI values for children decreased in the order of As > Pb > Hg > Ni > Cd > Cu > Cr > Zn, the HI values for adults decreased in the order of As > Pb > Hg > Cd > Cr > Ni > Cu > Zn, and the HI values for seniors decreased in the order of As > Pb > Hg > Cd > Cu > Cr > Ni > Cu > Zn.

The HI caused by As was the largest for all three age groups. Pb and Hg also had relatively large contributions to HI. The HIs for all age groups were less than 1, indicating that the non-carcinogenic risks were at a safe level.

**Figure 4:**
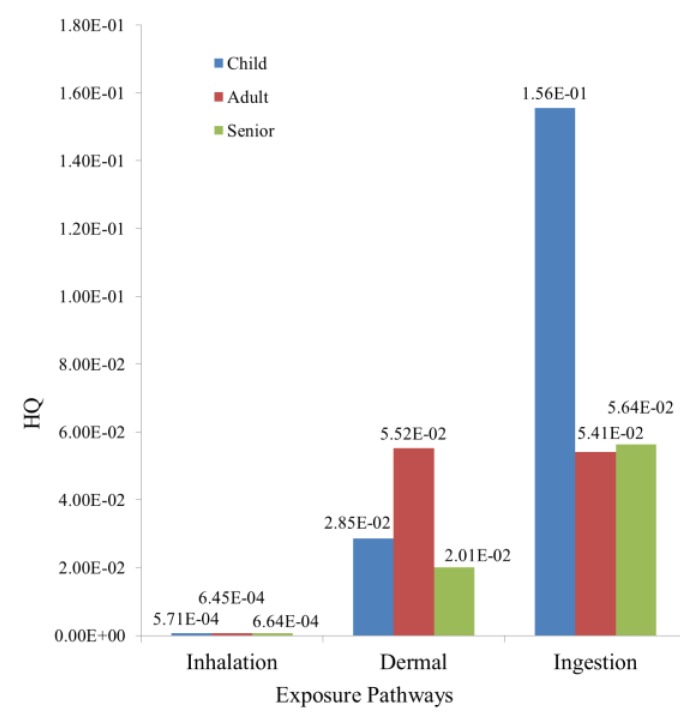
Hazard quotients (HQs) of different age groups under different exposure pathways.

With respect to the pathways of soil HM exposure, the HQ under inhalation exposure had the following decreasing order: seniors > adults > children; the HQ under dermal contact exposure had the following decreasing order: adults > children > seniors; the HQ under ingestion exposure had the following decreasing order: children > seniors > adults (Figure 4). The HQs caused by the ingestion of HMs for children were significantly greater than those for adults and seniors. The risk of soil ingestion was 10 times more than those of inhalation and dermal exposure; thus, this factor must be considered during the health risk assessment. These results indicated that As made the greatest contribution to the potential health risk for different age groups and that ingestion was the main threat pathway for citizens’ health in the study area, while the rest of the studied metals and pathways caused almost no non-carcinogenic risks.

#### 3.3.3. Carcinogenic Risk Assessment

**Figure 5:**
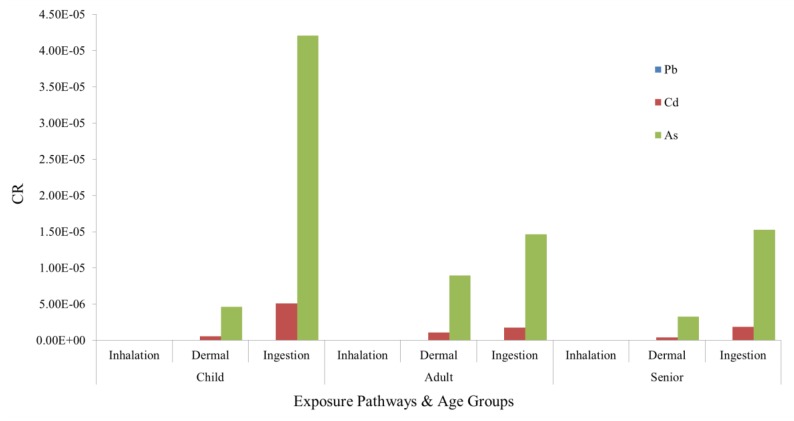
CRs of the different elements for different age groups under different exposure pathways.

Due to the lack of carcinogenic slope factors for Cr, Hg, Cu, Ni, and Zn, only the carcinogenic hazard indices for Pb, Cd, and As were estimated (Figure 5). With respect to the CRs of the soil HMs, the CR of As was the largest, followed by those of Cd and Pb. This might be related to the severe toxicity of As. It has been reported that exposure to two or more pollutants may result in additive and/or interactive adverse effects. Therefore, it was difficult to assess the potential health risks of multiple metals using each individual HQ value for the HMs.

**Figure 6:**
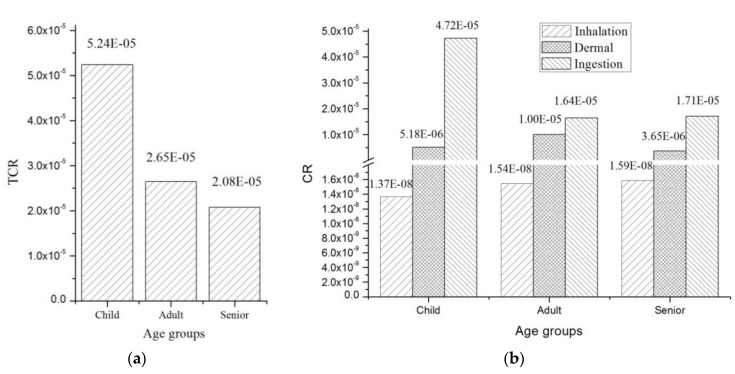
Probabilities of carcinogenic risk (CRs) (**a**) and total carcinogens risks (TCRs) (**b**) for different age groups under different exposure pathways.

The total carcinogens risks (TCRs) for different age groups are presented in Figure 6a. The TCR for children was the greatest (5.24 × 10^−5^), followed by those of adults (2.65 × 10^−5^) and seniors (2.08 × 10^−5^). In terms of the pathways of soil HM exposure, the components of different exposure pathways followed the order: ingestion > dermal contact > inhalation (Figure 6b).

The CRs of all age groups were less than the unacceptable level of 10^−4^ set by the USEPA but higher than the safety limit value of 10^−6^. Therefore, the potential carcinogenic risks for the exposure of local residents to HMs should not be overlooked.

### 3.4. Comparison of Heavy Metals in the Soil-Plant-Human System 

#### 3.4.1. Translocation from Soil to Plants

**Figure 7:**
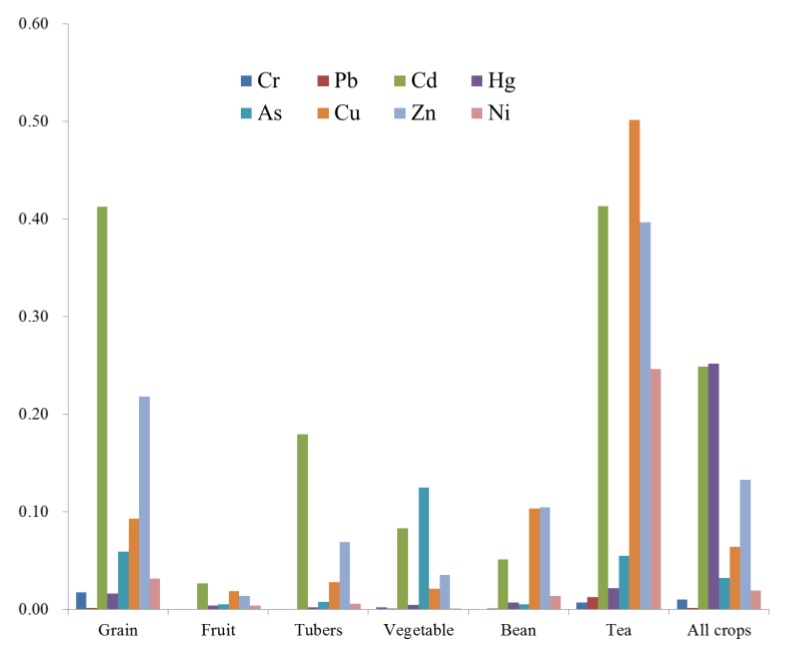
The bioaccumulation factors (BAFs) of heavy metals in different crops.

The BAFs of different HMs for different crops are presented in Figure 7. The overall BAF for all crops followed the order Cd (0.249) > Zn (0.133) > As (0.076) > Cu (0.064) > Ni (0.018) > Hg (0.011) > Cr (0.010) > Pb (0.001). As shown in Figure 7, Cd was most easily uptaken by crops while Pb was identified as having the lowest accumulation in crops.

Tea was observed to have the largest BAFs of HMs and fruit had the smallest BAFs of HMs (Figure 7). The largest BAF of grains was for Cd (0.4125), and the smallest was for Pb (0.001). Fruits and tubers also had the largest BAF for Cd (0.027 and 0.179, respectively). Vegetables had the largest BAF for As (0.125) and the smallest for Ni and Pb (0.001). Beans had the largest BAF for Zn (0.105) and the smallest for Cr and Pb (0.001). Tea had the largest BAF for Cu (0.501) and the smallest for Pb (0.001). The BAFs for Cd in almost all of the crops were very high compared with those of the other elements, indicating that Cd was more easily absorbed by the crops.

These results indicated that different crops have different capacities to absorb HMs in soil, so citizens can adjust their planting structure and spatial distribution of crops according to the distributions of the different elements. In areas with high HM contents in the soil and high HM enrichment coefficients, we can reduce the potential harm to human health by controlling the pollution source, regulating and adjusting agronomic measures, adjusting planting patterns, and changing land-use types.

#### 3.4.2. Comparison of Heavy Metal Pollution Risks in the Soil-Pant-Human System

**Figure 8:**
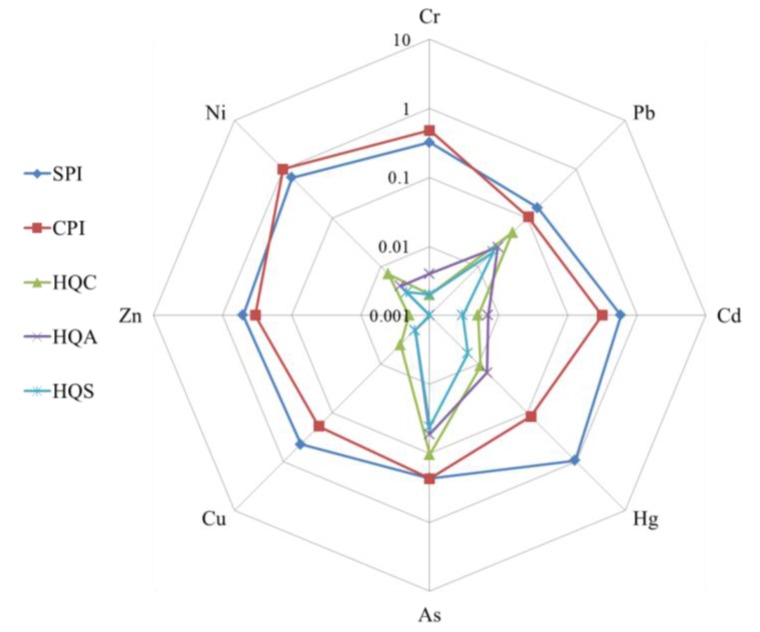
The radar sequence diagram of the mean SPI, CPI, and HQ for different heavy metals.

To analyse the differences in HM contamination in soil and plants and the health risks to humans, the radar sequence diagram of the mean values of SPI, CPI, and HQ for different HMs was plotted (Figure 8). SPI was used to assess the ratio of HM in the soil to its standard value. CPI was used to assess the ratio of HM in plants to its limit value. HQ was used to assess the ratio of non-carcinogenic risk due to exposure to HMs. HQC, HQA, and HQS represent the HQs for children, adults, and seniors, respectively. As shown in Figure 8, the SPIs for all the elements were less than 1.0 and larger than 0.1, representing overall conditions of relative safety. However, the mean HQ for Hg was 0.948, very close to the limit value of 1.0. This warns that the content of Hg in soil in the study area is likely to pose certain potential health risks to citizens. The CPIs for Cr, Pb, Cd, Hg, As, Cu, and Zn were less than 1.0 and larger than 0.1. However, the CPI of Ni was 1.007, indicating that the Ni concentration in crops was higher than the guideline value set in the national standards. All the HQs of the HMs for the different age groups were significantly less than the alert value 1.0 and were at a safe level.

**Figure 9:**
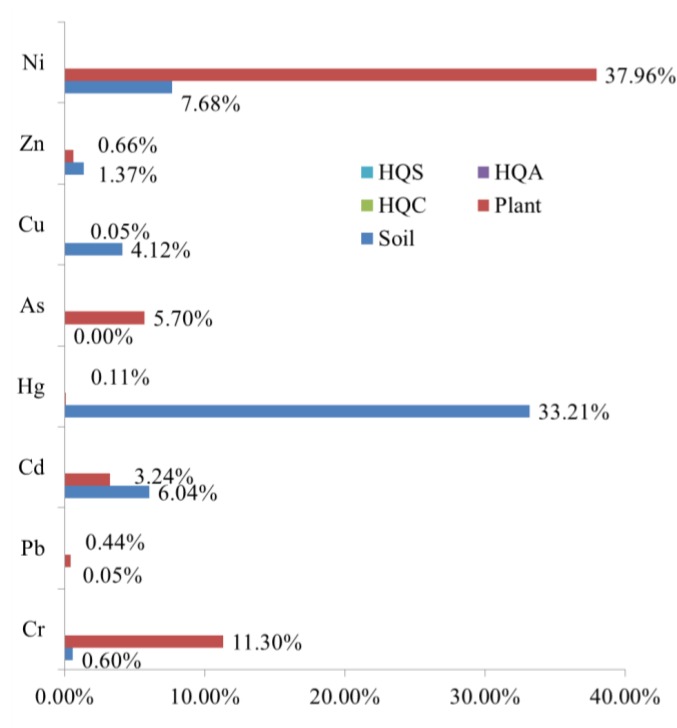
Comparison of the standard exceeding rates of SPI, CPI, and HQ for different heavy metals.

The standard exceeding rates of SPI, CPI, and HQ—and all HQs for children, adults, and seniors—for eight HMs were 0, showing that the non-carcinogenic risks in all samples were at the safety level. Great differences existed in the standard exceeding rates of HMs between soil and plants. As shown in Figure 9, the standard exceeding rates of Cu, Hg, and Cd in soil were significantly higher than the corresponding values in crops. Meanwhile, the standard exceeding rates of Ni, As, and Cr in crops were significantly higher than the corresponding values in soil.

From the investigated data, Hg is the most polluted trace HM in the soil, while Ni is the most polluted trace HM in the crops. This indicated that in addition to the content of HMs, some other factors also influenced the translocation of HMs from soil to plants. Therefore, in future research, more attention should be paid to the factors that affect the crops’ uptakes of heavy metals in soil. Additionally, more soil properties should be monitored and analysed to help us make clear what these factors are. Although some of the soil samples were polluted by HMs, the potential health risks of HMs for all samples and all age groups were at the safe level. This showed that farmers and consumers in the YRD region do not need to worry too much about food safety and their health.

## 4. Conclusions

The state of heavy metal pollution in the soil-plant-human system was analysed in a typical coastal industrial region in the YRD, and the assessment of non-carcinogenic and carcinogenic risk via three exposure pathways was employed. Finally, the BAF, representing the transformation of HMs from soil to plants, was calculated, and the discrepancies in the exceeding rates of SPI, CPI, and HQ for different heavy metals were analysed.

The mean contents of Cr, Pb, Cd, Hg, As, Cu, Zn, and Ni in soil were 69.64, 42.89, 0.20, 0.31, 6.67, 35.50, 111.16, and 29.99 mg/kg, respectively. The mean contents of Cr, Pb, Cd, Hg, As, Cu, Zn, and Ni in crops were 0.44, 0.05, 0.05, 0.02, 0.16, 1.97, 14.22, and 0.39 mg/kg, respectively. The mean CPIs were in the following decreasing order: Ni (1.007) > Cr (0.483) > Zn (0.335) > Cd (0.314) > As (0.232) > Cu (0.187) > Hg (0.118) > Pb (0.105). The standard exceeding rates of Cu, Hg, and Cd in soil were significantly higher than the corresponding values in crops while the standard exceeding rates of Ni, As, and Cr in crops were significantly higher than the corresponding values in soil.

Compared with inhalation and dermal contact as direct soil exposure, soil ingestion was the most significant contributor to the total health risk. Children had the greatest health risk of heavy metals in the soils followed by adults and seniors. This is consistent with previous research [36]. With respect to the CRs of soil HMs, the CR of As was the largest, followed by those of Cd and Pb.

The results indicated that different crops had different absorption capacities for HMs. The BAFs of all crops followed the order Cd (0.249) > Zn (0.133) > As (0.076) > Cu (0.064) > Ni (0.018) > Hg (0.011) > Cr (0.010) > Pb (0.001).

Further study should focus on the following issues:

(1) The bioaccumulation values rather than the total contents of HMs should be taken into consideration. (2) Dynamics of HMs in soil and their absorption by plants are strongly affected by soil properties, which play a central role in the in the bioaccessibility of HMs, as such, some other soil properties should also be considered in further research studies. (3) To better analyse the health risks, crops should be collected at their appropriate maturation states instead of at their corresponding maturation times.

## Figures and Tables

**Table 1:** Classes of the single pollution index (SPI).

Class	*SPI*	Grade	Description of Soil Heavy Metal Pollution
1	≤1.0	Safety	Clean
2	1.0 < *SPI* ≤ 2.0	Slight pollution	Slightly clean
3	2.0 < *SPI* ≤ 3.0	Mild pollution	Soil pollution exceeds background, crops start to be polluted
4	3.0 < *SPI* ≤ 5.0	Moderate pollution	Soils and crops have been polluted moderately
5	*SPI* > 5.0	Severe pollution	Soils and crops have been polluted severely

**Table 2:** Classes of the Nemerow composite pollution index (NCPI).

Class	*NCPI*	Grade	Description of Soil Heavy Metal Pollution
1	≤0.7	Safety	Clean
2	0.7 < *NCPI* ≤ 1.0	Alert	Slightly clean
3	1 < *NCPI* ≤ 2.0	Slight pollution	Soil pollution exceeds background, crops start to be polluted
4	2 < *NCPI* ≤ 3.0	Moderate pollution	Soils and crops have been polluted moderately
5	*NCPI* > 3.0	Severe pollution	Soils and crops have been polluted severely

**Table 3:** National hygienic standard value of heavy metal (HM) content in food in China (mg/kg).

Element	Food	Vegetables	Beans	Tubers	Fruits	Series of National Standard
Pb	0.4	0.2	0.8	0.4	0.2	GB14935-1994
Cd	Rice 0.2	0.05	0.05 ^a^	0.05 ^a^	0.03	GB15201-1994
	Flour 0.1					
	Coarse Cereals 0.05					
Hg	0.02	0.01	0.01 ^b^	0.01	0.01	GB2762-1994
Cu	10	10	20	20 ^c^	10	GB15199-1994
Cr	1.0	0.5	1.0	0.5	0.5	GB14961-1994
Zn	50	20	100	100 ^d^	5	GB13106-1991
As	0.7	0.5	0.5 ^e^	0.5 ^e^	0.5	GB4810-1994
Ni	0.4	0.3	0.3 ^f^	0.3 ^f^	0.2	[33]

^a^ Standard value of Cd in beans and tubers referenced to the corresponding value of vegetables; ^b^ Standard value of Hg in beans referenced to the corresponding value of vegetables, tubers, and fruits; ^c^ Standard value of Cu in tubers referenced to the corresponding value of beans; ^d^ Standard value of Zn in tubers referenced to the corresponding value of beans; ^e^ Standard value of As in tubers and beans referenced to the corresponding value of vegetables and fruits; ^f^ Standard value of Ni in tubers and beans referenced to the corresponding value of vegetables.

**Table 4:** Summary statistics of the heavy metal contents in soil (*N* = 1822).

Content	Cr (mg/kg)	Pb (mg/kg)	Cd (mg/kg)	Hg (mg/kg)	As (mg/kg)	Cu (mg/kg)	Zn (mg/kg)	Ni (mg/kg)
Mean	69.64	42.89	0.20	0.31	6.67	35.50	111.16	29.99
Median	71.10	42.65	0.18	0.21	6.38	33.45	107.00	30.40
Std	27.53	15.43	0.09	0.32	2.57	15.13	34.90	15.61
CV (%)	39.53	35.98	45.00	103.23	38.53	42.62	31.40	52.05
Min	9.16	15.60	0.03	0.02	0.88	7.14	34.30	3.81
Max	326.00	263.00	1.83	2.26	19.10	160.00	714.00	293.00
Background value	56.1	36.2	0.161	0.076	5.75	23.1	86.6	20.7
Critical value [46]	150	250	0.3	0.3	30	50	200	40

**Table 5:** Summary statistics of heavy metal contents in crops (*N* = 1822).

Content	Cr (mg/kg)	Pb (mg/kg)	Cd (mg/kg)	Hg (mg/kg)	As (mg/kg)	Cu (mg/kg)	Zn (mg/kg)	Ni (mg/kg)
Mean	0.44	0.05	0.05	0.02	0.16	1.97	14.22	0.39
Median	0.20	0.04	0.03	0.00	0.05	2.10	17.00	0.26
Std.	0.75	0.09	0.06	0.02	0.41	1.57	10.93	0.49
Skewness	5.50	9.66	5.16	2.277	8.15	1.86	0.13	4.53
Kurtosis	57.42	130.12	59.09	8.338	98.52	15.37	−1.29	44.94
CV (%)	170.45	180.00	120.00	120.80	256.25	79.70	76.86	125.64
Min	0.01	0.01	0.01	0.01	0.01	0.10	0.39	0.01
Max	13.00	1.50	1.10	0.024	6.80	21.00	56.00	7.80

**Table 6:** Descriptive statistics of SPIs and NCPIs (mg/kg, *N* = 1822).

Item	Cr	Pb	Cd	Hg	As	Cu	Zn	Ni	NCPI
Mean	0.321	0.162	0.585	0.948	0.229	0.438	0.508	0.670	0.846
Median	0.290	0.170	0.570	0.670	0.210	0.330	0.510	0.670	0.625
Std.	0.171	0.070	0.338	1.083	0.110	0.302	0.192	0.382	0.740
Min	0.040	0.040	0.100	0.030	0.030	0.050	0.170	0.100	0.150
Max	1.890	1.050	6.100	7.530	0.710	2.500	3.570	7.330	5.490
CV (%)	53.26	43.25	57.77	114.22	47.94	69.12	37.82	56.97	87.42

**Table 7:** Pollution grade classification of the SPIs in soil (*N* = 1822).

Pollution Degree	Cr (%)	Pb (%)	Cd (%)	Hg (%)	As (%)	Cu (%)	Zn (%)	Ni (%)
Safety	99.40	99.95	92.86	66.68	100	95.88	98.63	92.04
Slight pollution	0	0.05	6.75	20.09	0	3.90	1.26	7.24
Mild pollution	0	0	0.27	6.59	0	0.22	0.05	0.27
Moderate pollution	0	0	0	5.76	0	0	0.05	0.33
Severe pollution	0	0	0.11	0.88	0	0	0	0.11
Polluted	0.60	0.05	7.14	33.32	0	4.12	1.37	7.96

**Table 8:** Descriptive statistics of the crop pollution index (CPI) (mg/kg, *N* = 1822).

Item	Cr	Pb	Cd	Hg	As	Cu	Zn	Ni
Mean	0.483	0.105	0.314	0.118	0.232	0.187	0.335	1.007
Median	0.260	0.077	0.235	0.073	0.070	0.190	0.380	0.700
Std	0.746	0.167	0.337	0.128	0.587	0.149	0.277	1.119
Min	0.000	0.000	0.000	0.004	0.001	0.001	0.003	0.006
Max	13.000	3.250	5.500	1.43	9.714	2.100	5.800	13.500
CV (%)	154.50	158.76	107.56	108.20	252.68	79.95	82.62	111.15
Number of polluted samples	206	8	59	2	104	1	12	692
Percent of polluted samples	11.30	0.44	3.24	0.11	5.70	0.05	0.66	37.96
